# Observation of human-animal interaction for research (OHAIRE) behavior coding in a randomized control trial of children with attention-deficit hyperactivity disorder (ADHD) and a canine-assisted intervention

**DOI:** 10.3389/fpsyt.2024.1327380

**Published:** 2024-02-27

**Authors:** Leanne O. Nieforth, Noémie A. Guerin, Annamarie Stehli, Sabrina E. B. Schuck, Katherine Yi, Marguerite E. O’Haire

**Affiliations:** ^1^Center for the Human-Animal Bond, College of Veterinary Medicine, Comparative Pathobiology, Purdue University, West Lafayette, IN, United States; ^2^Implicity, Paris, France; ^3^School of Medicine, University of California Irvine, Irvine, CA, United States; ^4^College of Veterinary Medicine, University of Arizona, Tucson, AZ, United States

**Keywords:** animal-assisted intervention, therapy dog, attention-deficit hyperactivity disorder, complementary intervention, human-animal interaction

## Abstract

**Introduction:**

Diagnosed in about 10% of children in the United States, attention-deficit hyperactivity disorder (ADHD) is characterized by symptoms including inattention, hyperactivity, and impulsivity. Traditional interventions, such as pharmacological and psychological interventions, are often used in conjunction with integrative health options, such as animal-assisted interventions. The objective of this manuscript is to report behavior coding findings from a randomized control trial of children with ADHD.

**Methods:**

As part of a larger randomized control trial focused on the efficacy of combining a canine-assisted intervention (live therapy dog or control stuffed dog) with cognitive behavioral therapy for children with ADHD, the current manuscript focuses on video-captured behavior observations (n = 35 children, approximately 322 minutes of data). Data were extracted and coded using the Observation of Human-Animal Interaction Research (OHAIRE) Coding System. Behavior codes are reported as summary scores for the following domains: animal social interaction and human social interaction (further separated into human-adult social interaction and human-peer social interaction). Repeated measures mixed models analyses were performed using SAS PROC GLIMMIX to evaluate group differences and change across the study period.

**Results:**

There were no significant differences in how much children interacted with the live therapy dogs versus control stuffed dogs. With respect to human-to-human social interactions, children showed greater increases over time in human-directed social interactions in the presence of live therapy dogs compared to stuffed dogs (p = .020). Over the course of the 12-week intervention, children increased in interactions with both adults (p = .006) and their peers (p = .014); however, there were more increases over time in adult-directed social interactions in the live animal condition compared to the control stuffed animal condition (p < 0.0001).

**Discussion & conclusions:**

Findings suggest changes in social interaction when participating in this canine-assisted intervention, specifically greater increases in human-to-human social interactions over time when a live therapy dog is present compared to a control stuffed dog. Children appear to engage relatively equally with both live and stuffed dogs; however, the impact of animals on human socialization differs based on if a live animal is present. Future studies should consider incorporating behavior coding analysis into studies of canine-assisted interventions to identify how human-animal interactions may be moderators or mechanisms for psychosocial outcomes.

## 1 Introduction

Attention deficit hyperactivity disorder (ADHD) is characterized by inattention and hyperactivity or impulsivity that is maladaptive, inconsistent with development, and has been present for at least six months (1). Data from a 2016-2019 survey suggests that 9.8% of children and adolescents in the United States have received a diagnosis of ADHD in their lifetime (2). The Center for Disease Control and Prevention recommends both pharmacological treatment and behavior therapy for ADHD (3). Pharmacological treatment is the administration of stimulants, non-stimulants, or antidepressants to relieve ADHD symptoms (4). Though pharmacological treatment is effective for some individuals, an estimated 21% of children discontinue ADHD medication due to negative side effects and perceived ineffectiveness (5). Behavior therapy covers a variety of psychosocial interventions, including but not limited to behavioral interventions facilitated by parents and teachers, cognitive therapy or neurological training, and one-to-one counseling (6).

Multimodal interventions have been demonstrated to decrease psychopathology and increase quality of life in individuals with ADHD (7). Multimodal interventions can include combining multiple types of traditional interventions or combining traditional interventions with complementary and integrative health interventions. Families may choose to use complementary and integrative health interventions for a variety of reasons (e.g., due to comorbid disorders, desire to try anything, or fear of adverse events of pharmacological interventions). The percentage of families that have tried complementary and integrative health options for ADHD treatment ranges from 5-64%, with the large range due to the inclusion of dietary changes as a complementary intervention option (8, 9). Common types of complementary interventions include vitamins and dietary supplements, herbal medicines, sensory integration, art, relaxation, neurofeedback, and massage (10).

Another type of complementary intervention, that is increasingly common, is an animal-assisted intervention (AAI). AAI is the partnership with an animal in any part of the intervention process and may include animal-assisted therapy (AAT), animal-assisted activities (AAA), animal-assisted education (AAE) or assistance animals (11). Anecdotally, individuals find these complementary options helpful, but there is still little clinical evidence, and the evaluation of the safety of the intervention is weak (12). To date, there have been only five randomized controlled studies conducted that have investigated the interaction between therapy animals and individuals with ADHD (13–17). Only two of these randomized controlled studies examine canine-assisted interventions while the others focused on equine-assisted interventions and farm animal experiences.

One randomized control study was a four-year study on public school special education students with ADHD (n = 26), autism spectrum disorder (ASD), and emotional disability (ED) (13). Children were bussed to a farm once a week for a two-hour session where they worked to gain a collection of skills to handle the different types of farm animals independently. During the first year of the study, the classroom teacher and farm teacher each evaluated the children on the Achenbach Teacher Rating System twice throughout the year. For the next three years, the teachers switched to the Behavior Assessment System for Children Teacher Rating System (BASC TRS). Results suggest that all BASC TRS factor scores and problem scores were lower in the farm program compared to the classroom, but the adaptability scores had not changed.

A second randomized study looked at physiological reactions to dogs in 17 children with ADHD (17). Systolic blood pressure (SBP), diastolic blood pressure (DBP), and heart rate were measured both during the control session (no dog present) and the experimental session (dog present- children given no instruction regarding interaction with dog). Teachers also rated the children’s behavior after each session on a five-point scale. Results demonstrate that there were no significant changes in teacher ratings. DBP significantly increased while children held the dog, SBP significantly increased following holding the dog, and heart rate significantly decreased following holding the dog. Findings suggest that the increase in blood pressure was interpreted as a response to the positive stimuli and that the decrease in heart rate was potentially an orienting behavior.

A third study was a randomized block design study that examined effects of hippotherapy (intervention group) versus a physical rehabilitation activity program (control group) on brain function of individuals with ADHD (14). Measures included physical characteristics, muscular and cardiorespiratory fitness, functional MRI, and brain-derived neurotrophic factor. Findings suggest that participation in hippotherapy significantly decreased body fat and increased brain-derived neurotrophic factor (14).

A fourth study, compared hippotherapy to pharmacotherapy for the treatment of ADHD (15). Measures included ADHD Rating Scale, Child Behavior Checklist, Self-esteem Scale, Pediatric Quality of Life Inventory, Developmental Coordination Disorder Questionnaire, Clinical Global Impressions-Severity and Electroencephalography. Results suggest improvement in ADHD symptoms and Clinical Global Impressions-Severity in both groups. The hippotherapy group also demonstrated improved attention, impulsivity/hyperactivity, and quality of life.

A fifth study, of particular interest as the parent study of the current project, a combination of cognitive behavioral therapy (CBT) and canine-assisted intervention (CAI) was investigated (16, 18, 19). In this study, 88 children with ADHD participated either in the control group (CBT without CAI) or experimental group (CBT and CAI). Parents completed the ADHD – Rating Scale – Fourth Edition, Home, and School Version, the Social Skills Improvement System – Rating Scales, Parent Form and Social Competence Inventory (18). Total ADHD symptoms, inattention and social skills had significant main effects related to group (16). There were also significant interaction effects (group x time) for problem behaviors and social initiation (16). Additionally, self-reported behavioral conduct, scholastic and social competence were significantly higher in the CAI group post-treatment than they were pre-treatment with no pre- to post- treatment changes in the non-CAI group indicating benefits to the intervention (19). The manuscript cited here consists of the intervention primary outcomes of the parent study. The current project is an extension of these findings, looking specifically at identifying if behaviors and interactions were different within the sessions themselves.

Taken together, these five studies align with one another in that they all suggest preliminary benefits for animal-assisted interventions for individuals with ADHD. Each study incorporates a different methodology, together suggesting both psychosocial and physiological improvements upon participation in an animal-assisted intervention. Though these five studies set the foundation for rigorous, empirical research incorporating multiple methodologies, additional forms of measurement, beyond surveys and physiological measures, are necessary to continue to build the evidence-base and understand the mechanisms occurring in the intervention. Specifically, there is a gap in the literature regarding the understanding of what is actually occurring between the children, the animals and the other humans present during the interaction. This is important to understand mechanistically how the intervention works. One methodology that has yet to be used in the examination of animal-assisted interventions for ADHD, yet will directly address this gap, is behavior coding. Behavior coding enables direct observation of changes in behavior during an intervention, providing an objective, empirical perspective of the intervention (20). The Observation of Human-Animal Interaction for Research (OHAIRE) Coding System is a standardized, validated behavior coding tool developed to measure the social interaction behaviors of participants, peers, and animals during both animal-assisted interventions and control conditions (21). The OHAIRE Coding System has demonstrated a convergence between OHAIRE recorded social behaviors and social skills assessed by the Social Skills Rating System (21). This convergence makes this particular coding system well-suited for this study as findings from the parent study suggest changes based on the Social Skills Rating System (19, 22). Previous studies incorporating behavior coding have been successful in objectively analyzing social behaviors of children with ADHD but have yet to incorporate coding of interactions between humans and animals (23, 24). Coding the social interactions between humans and animals is particularly relevant as a potential mechanism for social skill development in canine-assisted interventions (18, 22).

The purpose of the current manuscript is to report on the behavior coding of video data collected as part of the Schuck et al., 2018 randomized control trial. To date, this is the first manuscript to report on video recorded behavior coding in a randomized control trial of children with ADHD and a canine-assisted intervention. The hypothesis was that the presence of an animal within a canine-assisted intervention would lead to an increase in social behaviors both over time and between groups.

## 2 Methods

### 2.1 Study design

This manuscript is part of the Project Positive Assertive Cooperative Kids (P.A.C.K.) randomized control trial focused on examining a canine-assisted intervention combined with cognitive-behavioral therapy for children with ADHD. At the time of funding (2010), the parent institution did not approve of the trial being registered as a clinical trial as it had no medical devices or medicines being studied. Instead, the institution deemed it a randomized controlled trial. This study was approved by two University Institutional Review Boards (UC Irvine Protocol # 2010-7679, Purdue University Protocol # 1410015340) and received an exemption from Purdue Institutional Animal Care and Use Committee as the researchers did not have any interaction with the animals.

Eligibility for participation was determined from a screening procedure which included a parent-reported family medical and psychosocial history questionnaire, researcher administered Kaufman-Schedule for Affective Disorders and Schizophrenia for School-Age Children: Present and Lifetime Version, researcher administered Wechsler Abbreviated Scale of Intelligence, Second Edition and a semi-structured clinical-administered interview with parents and children based on the Diagnostic and Statistical Manual of Mental Disorders for psychiatric disorders (18). To be included in the study, participants had to have a primary diagnosis of ADHD, Combined subtype, be 6-9 years old, and have an estimated full scale IQ score of 80 or above and the ability to complete all screening measures (18). Participants were excluded if they were currently using medication for ADHD, had a diagnosis of a pervasive developmental disorder/autism, depression, anxiety, or epilepsy, or a history of animal cruelty (18). After participants were screened and eligible, informed consent was collected.

Participants completed a variety of clinical survey measures immediately prior to the study, during the study, and immediately following the study and 6-weeks after the 12-week intervention. Parents also completed surveys regarding symptom severity, social skills, and problem behaviors at the same timepoints. Clinical survey measures and associated outcomes can be found in (16) and (19).

Participants were randomly assigned to the canine assisted intervention group (registered live therapy dog) or the control group (toy stuffed dog). In addition to randomization, half of participants were placed in a waitlist condition to control for the possible influence of time and child development (18). All participants participated in a cognitive behavioral therapy intervention curriculum. The intervention curriculum, P.A.C.K., included components of the University of California, Irvine Child Development Center School-based Social Skills model, the Kids Interaction with Dogs Safely program and the Intermountain Therapy Animals’ Reading Education Assistance Dogs Program. Example activities included writing in journals, reading, and learning about different coping mechanisms.

Children participated in the study for 12 weeks for a total of 23 sessions. Three canines were part of the canine-intervention group, each partnered with a human handler who facilitated the interactions. There was a 1:2 ratio of dog or stuffed dog to children. Sessions were completed in large groups with multiple dogs available per session. In addition to the outcomes collected via screening interviews and study surveys, all sessions were video recorded to capture behavior observation data. The video-recording component of the study was an ancillary component that was added after the start of the trial. All participants were subject to the same randomization procedure, but since the recordings did not start at the beginning of the trial, fewer participants were included in this component of the study. The current manuscript explores the video-captured behavior observation components of the study.

### 2.2 OHAIRE coding procedure

Five sessions (sessions 1,7,12,18, and 23) were video recorded to capture behavior observation data. These sessions were selected to maximize the total number of participants present during the video recorded sessions and to represent sessions throughout the entirety of the study. Data extraction replicated the OHAIRE Coding System (25) where 10-minute video segments were divided into thirds and 1-minute segments were randomly selected from each of those segments. Therefore, three minutes from each of the selected sessions were randomly selected for behavioral coding for each participant. The OHAIRE coding system was specifically designed for human-animal interaction research projects and demonstrates good reliability and validity (21).

Two research assistants coded the behavior of children with ADHD and their peers. Coders were blinded to the aims and hypotheses of the study, but due to the nature of the study (presence of the dog vs. no dog) raters were not blind to the condition. Coder 1 coded 98% of the data (the 3 minutes that were not coded were dropped from the analysis) and coder 2 coded 23% of the data to establish interrater reliability. Interrater reliability was calculated using Cohen’s Kappa. The overall agreement among raters was 86.5% (k = .865, p<.001). Interrater reliability was also calculated for specific categories of interactions (k = .736, p<.001), facial emotional displays (k = .756, p<0.001), verbal valence (k = 0.98, p<0.001), social communication (k = .655, p<0.001), and problem behaviors (k = .894, p<0.001).

Across the study period’s five assessment sessions, a total of 322 minutes were selected for coding (intervention group: 173 minutes, control group: 149 minutes). The average per child was 9.2 minutes (SD: 2.66, Range: 3-13 minutes). Following the OHAIRE Coding System, coders rated the absence or presence of a behavior in 10 second intervals. The score for each behavior code is the number of 10-second intervals it was present within a minute (i.e., a count ranging from 0-6). Summary scores were created for the following domains: Animal Social Interaction and Human Social Interaction (further separated into Human-Adult Social Interaction and Human-Peer Social Interaction). Each summary score was the sum of the six “interactions” items towards the relevant target, resulting in variables with a possible range of 0-36.

### 2.3 Analysis

Demographics and baseline psychosocial measures of each group (dog vs. stuffed dog) were compared using independent sample t-tests for continuous measures and Fisher’s exact tests for categorical variables. Descriptive statistics, (i.e., means, variance, frequency distribution/quantiles) of each outcome measure were reviewed both across and within the sessions.

Repeated measures mixed models analyses were performed to evaluate group differences (dog versus stuffed dog), change across the study period and the group by time interaction. Utilizing PROC GLIMMIX in SAS, a random effects Poisson model with a loglink function was specified due to the count data. If there was overdispersion, a negative binomial regression model was implemented instead. The mixed model approach allowed for within subject intercorrelation due to repeated measures to be accounted for, and all participants to be included regardless of missed sessions, Individual participants contributed up to 3 ratings per session. Sensitivity analyses were performed to determine there were no significant biases related to those with more ratings in a session or with missing data for a given session.

## 3 Results

Final analysis included observational data from n = 35 participants (**Table 1**). There were no significant effects for any sociodemographic characteristics across groups (all p’s > 0.52). The intervention (CAI) and control (stuffed dog) groups did not differ by sex (p = 0.73), gender (p > 0.999), age (p = 0.68), grade (p = 0.64), ethnicity (p = 0.72), or race (p = 0.58). Additionally, scores from the ADHD-Rating Scale demonstrated no significant difference in ADHD symptom severity between groups (p =0.52-0.99).

**Table 1:** Demographics.

Characteristic	Dog, N = 18 (51%)	Control, N = 17 (49%)	Overall, N = 35	p-value^1^
**Sex, n (%)**				0.725
F	5 (28%)	6 (35%)	11 (31%)	
M	13 (72%)	11 (65%)	24 (69%)	
**Gender, n (%)**				> 0.999
Female	5 (28%)	5 (31%)	10 (29%)	
Male	13 (72%)	11 (69%)	24 (71%)	
Unknown	0	1	1	
**Age, n (%)**				0.675
6	0 (0%)	1 (5.9%)	1 (2.9%)	
7	11 (61%)	8 (47%)	19 (54%)	
8	5 (28%)	5 (29%)	10 (29%)	
9	2 (11%)	3 (18%)	5 (14%)	
**Grade, n (%)**				0.644
1	4 (24%)	5 (29%)	9 (26%)	
2	8 (47%)	5 (29%)	13 (38%)	
3	3 (18%)	6 (35%)	9 (26%)	
4	2 (12%)	1 (5.9%)	3 (8.8%)	
Unknown	1	0	1	
**Ethnicity, n (%)**				0.721
Hispanic/Latino	7 (41%)	5 (31%)	12 (36%)	
Non-Hispanic	10 (59%)	10 (62%)	20 (61%)	
Decline to Answer	0 (0%)	1 (7%)	1 (3%)	
Unknown	1	1	2	
**Race, n (%)**				0.582
Alaska Native	0 (0%)	0 (0%)	0 (0%)	
Asian	2 (12%)	3 (18%)	5 (15%)	
African American	0 (0%)	0 (0%)	0 (0%)	
Pacific Islander/Native Hawaiian	0 (0%)	0 (0%)	0 (0%)	
Caucasian	10 (59%)	9 (53%)	19 (56%)	
Multiple	5 (29%)	3 (18%)	8 (24%)	
Other	0 (0%)	2 (12%)	2 (5.9%)	
Unknown	1	0	1	
**ADHD Symptoms Baseline, Mean (SD)**	13.33 (3.65)	13.06 (3.59)	13.21 (3.57)	0.808
Unknown	0	1	1	
**Hyperactive Impulsive Symptoms Baseline, Mean (SD)**	6.06 (2.69)	6.12 (2.28)	6.09 (2.47)	0.875
Unknown	0	1	1	
**Inattention Symptoms Baseline, Mean (SD)**	7.28 (2.19)	6.94 (2.21)	7.12 (2.17)	0.523
Unknown	0	1	1	
**ADHD Total Score Baseline, Mean (SD)**	36.17 (7.73)	34.69 (6.95)	35.47 (7.30)	0.545
Unknown	0	1	1	
**Hyperactive Impulsive Frequency Baseline, Mean (SD)**	16.83 (4.96)	16.19 (4.25)	16.53 (4.58)	0.665
Unknown	0	1	1	
**Inattention Frequency Baseline, Mean (SD)**	19.33 (4.45)	18.50 (4.35)	18.94 (4.36)	0.579
Unknown	0	1	1	
**ODD Symptoms Baseline, Mean (SD)**	3.28 (2.97)	3.25 (2.89)	3.26 (2.88)	0.986
Unknown	0	1	1	
**ODD Total Score Baseline, Mean (SD)**	11.11 (6.21)	10.44 (6.91)	10.79 (6.46)	0.653
Unknown	0	1	1	

^1^Fisher’s exact test; Wilcoxon rank sum test.

Both pre/post models and summary models including all session assessment points were conducted. Both follow the same patterns, so the summary models inclusive of all assessment points are presented here (**Table 2**).

**Table 2:** Model results: summary scores.

	F Value	Pr > F
Animal Interaction		
Condition	0.47	0.496
Session	0.03	0.873
Session*Condition	0.66	0.416
Human Adult Interaction		
Condition	3.44	0.065
Session	8.81	0.006
Session*Condition	16.93	< 0.001
Human Peer Interaction		
Condition	0.88	0.348
Session	6.73	0.014
Session*Condition	0.09	0.767
Human Total Interaction		
Condition	0.79	0.376
Session	0.19	0.667
Session*Condition	5.53	0.020

The * indicates the interaction.

The Animal Social Interaction summary model found nonsignificant effects across conditions (p = 0.496), sessions (p = 0.873) and within the group x time interaction (p = 0.416). The Human Social Interaction summary model found a nonsignificant session effect (p = 0.667), a nonsignificant condition effect (p = 0.376), and a significant group x time interaction (p = 0.020). Given the significant interaction effect within the human interaction model, further exploration was conducted to examine human-adult versus human-peer interactions. The Human-Adult Social Interaction model found a nonsignificant condition effect (p = 0.065), but a significant session effect (p = 0.006) and a significant group x time interaction effect (p < 0.0001). The Human-Peer Social Interaction model found a nonsignificant condition effect (p = 0.348), a significant session effect (p = 0.014) and a nonsignificant group x time interaction effect (p = 0.767).

Findings suggest a different pattern of Human Social Interaction over time across the treatment versus control group. Specifically, human-directed social interaction increases more over time when a live dog is present compared to a stuffed dog. With respect to social interactions with children and adults, both increase over the course of the intervention program. The significant interaction in Adult Social Interaction indicates that the change over time differs between groups (e.g., potentially that adult interactions increase more over time in the live dog group).

## 4 Discussion

To our knowledge, this manuscript is the first to employ behavior coding to assess the interactions of children with ADHD and animals during a structured AAI. Although interactions with animals are similar in both groups (live and stuffed animal dogs), results show change in participant interactions with humans, most saliently with adults. Individuals diagnosed with ADHD may have difficulties in social interactions (26). Previous human-animal interaction research suggests that animals may be a social facilitator or an external focus of attention that may have positive impacts on social interactions (27, 28). These findings align with the quantitative survey findings from this same randomized controlled trial study, showing that structured canine-assisted interventions not only increase self-reported behavioral conduct, scholastic competence and social competence, but may also promote social interaction for children with ADHD (16). Given that both adult interaction and peer interaction increased over the course of the intervention, yet the change over time differed between these, practitioners should consider how opportunities to interact socially are intentionally integrated into canine-assisted interventions or animal-assisted interventions more broadly. Highlighting opportunities to engage with peers and adults or incorporating the guidance of an adult into peer-to-peer interaction (or vice versa) may identify ways to refine the intervention focusing on the potential benefits of the intervention in providing increased interactions between participants and other individuals present, whether children or adults.

Findings also highlighted that the children in the current study socially interacted in a similar format and frequency with both live and stuffed dogs. It may be that the theme of dogs or representation of dogs enables similar social interaction patterns as live dogs. Multiple studies have compared live animals to stuffed animals in the context of AAI and outcomes are mixed. For example, research examining the activation brain activity suggests that both interaction with a live dog and a stuffed animal dog increased brain activity, but the live dog stimulated more activity than the stuffed dog (29). Another study suggests that interaction with a robot dog and a live dog can be similar regarding the effects on mood, but different when examined on a deeper cognitive attribution level (30). Other research suggests differences in live dog versus stuffed dog regarding children laughing more, keeping their gaze on the dog, and increased social interactions with the live dog in comparison to the stuffed dog or control toy (31). Considering our findings within this larger body of work suggests that there are multiple mechanisms affecting these interactions. Our findings suggest that the frequency of interaction with the source (live or stuffed dog) may not be driving the changes in outcome differences between live and stuffed dogs because those frequencies are similar between groups. There is potentially another mechanism at play, highlighting that there is something else about a live animal that drives the changes in outcomes, beyond the frequency of social interactions. Additional studies are needed to identify this mechanism or group of mechanisms.

A few limitations should be considered regarding the results presented. First, this study included only one population of children with ADHD and had a small sample size. Second, only select sessions within the intervention were video recorded. Given the manualized nature of the intervention, this may have affected the behaviors that appeared in the dataset. For example, the structured intervention protocol directs adults and children to engage in certain activities (e.g., sitting and listening) rather than free, open-ended interactions in many cases. This would limit the availability and variability of some social behaviors (e.g., talking and playing) during specific sessions. Although these structured formats were equivalent across the treatment and control groups, they may have limited the time available to observe behavioral variation across participants. Given that the OHAIRE Coding tool was designed to assess unstructured interactions, results may have varied if the sessions included more opportunities to help the animal or touch the animal based upon the protocol. Similarly, if peer-interaction is a focus of the program, creating opportunities to help peers within the canine-assisted intervention may have altered the behaviors observed. Future studies should consider adapting the OHAIRE Coding tool to incorporate overarching program goals and to modify it to the specific animal species and programmatic goals of interest. Tailoring the structure to the needs of the animal (i.e., teaching participants to recognize and address the needs of the animal within the intervention) may positively promote the welfare of the animals included in the intervention.

## 5 Conclusion

The purpose of the current manuscript was to report on findings from video-recorded behavior coding in a randomized control trial of children with ADHD and a canine-assisted intervention (16). The hypothesis was that the presence of an animal within a canine-assisted intervention would lead to an increase in social behaviors. Participants demonstrated greater increases in human-directed social interaction over time in the live therapy dog condition, compared to the control stuffed dog condition. While interactions with peers and adults increased over time in both conditions, changes were more salient for adult interactions in the live therapy dog condition. Interestingly, there were no significant findings regarding differences in the interaction with the animals between groups, suggesting no differences in the frequency of interaction with a live dog versus a stuffed dog. Results are preliminary but suggest potential benefits of canine-assisted interventions for social interaction patterns in children with ADHD.

## Data availability statement

The raw data supporting the conclusions of this article will be made available by the authors, without undue reservation.

## Ethics statement

The studies involving humans were approved by Purdue University Institutional Review Board (#1410015340) and UC Irvine Institutional Review Board (# 2010-7679). The studies were conducted in accordance with the local legislation and institutional requirements. Written informed consent for participation in this study was provided by the participants’ legal guardians/next of kin.

## Author contributions

LN: Data curation, Formal analysis, Methodology, Writing – original draft, Writing – review & editing. NG: Investigation, Writing – original draft, Writing – review & editing. AS: Formal analysis, Methodology, Software, Writing – review & editing. SS: Conceptualization, Funding acquisition, Investigation, Methodology, Project administration, Resources, Supervision, Writing – review & editing. KY: Data curation, Writing – review & editing. MO: Conceptualization, Investigation, Methodology, Project administration, Resources, Supervision, Writing – review & editing.
